# Effects of BL Exposure on Circadian Rhythms and Rhythm-Related Disorders

**DOI:** 10.3390/ijms27219893

**Published:** 2026-11-04

**Authors:** Xiaoli Wang, Xiaoqi Pei, Chiyu Ge, Jiaqi Chen, Jing Xu, Jialin Wu, Hongji Lu, Lu Xu, Huandi Weng, Xiaoyin Chen, Li Deng, Huachong Xu

**Affiliations:** 1 School of Traditional Chinese Medicine, Jinan University, Guangzhou 510632, China; xiaoli_w666@163.com (X.W.); peixiaoqi@stu2024.jnu.edu.cn (X.P.); gcy0812@stu2024.jnu.edu.cn (C.G.); jiaqichen0728@163.com (J.C.); wujialin@stu2020.jnu.edu.cn (J.W.); luhji@stu.jnu.edu.cn (H.L.); luxu816@jnu.edu.cn (L.X.); dengli@jnu.edu.cn (L.D.); 2 School of Traditional Chinese Medicine, Guangdong Pharmaceutical University, Guangzhou 510632, China; xuj_0123@163.com; 3 Guangdong Provincial Key Laboratory of Syndrome and Formula, School of Pharmaceutical Sciences, Guangzhou University of Chinese Medicine, Guangzhou 510006, China; wenghuandi@foxmail.com; 4 Guangdong Provincial Key Laboratory of Traditional Chinese Medicine Informatization, Jinan University, Guangzhou 510632, China; 5 Key Laboratory of CNS Regeneration, Ministry of Education, Jinan University, Guangzhou 510632, China

**Keywords:** rhythm disruption, light exposure, sleep disruptions, neurodegenerative diseases, BL therapy

## Abstract

Circadian rhythms represent a core physiological mechanism sustaining human physiological homeostasis and normal bodily function. Environmental light serves as a primary exogenous *zeitgeber* for circadian regulation. Among visible light spectral components, BL (BL) exerts a critical modulatory influence on the circadian system, with dual implications for physiological regulation and clinical intervention: excessive or inappropriately timed exposure may lead to circadian disruption, whereas appropriately timed BL interventions show potential to improve circadian alignment in selected populations. Accumulated evidence indicates that circadian disruption is associated with sleep disorders, mood disturbances, neurodegenerative disorders, as well as metabolic and immune dysfunction, and may have bidirectional relationships with disease progression. Nevertheless, most existing studies concentrate on circadian modulation by broad-spectrum light. Few efforts have synthesized the molecular mechanisms, potential disease-related pathways, and targeted intervention frameworks of BL-associated circadian disruption, and the translational potential of BL’s bidirectional effects remains under-summarized, forming a prominent research gap. To fill this gap, this narrative review centers on the “BL-circadian rhythm-disease” regulatory axis. It outlines biological pathways in which circadian disruption may be implicated in multiple neurodegenerative disorders, highlights the circadian-modulating properties of BL, describes evidence linking inappropriate BL exposure to circadian disruption and disease onset or progression, and compiles clinical findings on BL-based interventions and their potential benefits for circadian function and selected disease-related symptoms. However, much of the evidence derives from observational studies and animal experiments, and a causal pathway linking BL exposure to disease through circadian disruption has not been definitively established. This review discusses the potential adverse and beneficial effects of BL in circadian disruption-related pathologies, addresses current research limitations, and offers theoretical references and fresh insights for mechanistic investigations, clinical research, and translational development of BL-targeted interventions.

## 1. Introduction

Rhythm disruptions are gaining attention as important health factors. The underlying mechanisms are diverse, and light conditions, as extremely critical factors, play a non-negligible role in rhythmic disruptions. From an evolutionary perspective, light, as an important environmental factor, plays an indispensable role in the regulation of biological rhythms. Early studies revealed an elevated rate of depression among polar region residents during winter light deprivation, confirming the effect of light on mood disruptions following circadian disruption [1,2]. With the rapid development of modern society, the popularization of electronic devices and the wide application of light-emitting diode (LED) lights, while convenient, have significantly changed the human light environment, resulting in increasingly serious circadian disruption problems that exert a profound impact on physiological rhythm. Studies have shown that the light emitted by electronic devices and LED light sources, especially BL, is a key factor affecting the circadian rhythm. Excessive use of electronic devices at night or prolonged exposure to LED light not only disrupts the normal function of the retina, but also leads to a decrease in cognitive ability and alertness the next day, while interfering with sleep structure [3,4,5]. These findings reveal multiple negative effects of circadian disruption on organisms and suggest that BL plays a key role in inducing circadian disruption and related diseases.

Although inappropriate light exposure can disrupt circadian rhythms, the ability of light to modulate circadian timing also provides a rationale for its therapeutic use. Light-based interventions therefore hold promise for disorders associated with circadian dysfunction. For example, blue-enriched light therapy improved circadian activity rhythms and motor performance in two mouse models of Huntington’s disease (HD) [6]. Building on these findings, this review examines the links between inappropriate light exposure, circadian disruption, and disease, and evaluates the evidence for and therapeutic potential of light-based interventions. This synthesis may help clarify the health implications of modern light exposure patterns and inform the development of interventions targeting circadian dysfunction.

## 2. Literature Search and Selection

A comprehensive literature search was conducted using the PubMed database to identify relevant articles published up to September 2026. Search terms included “BL”, “circadian rhythm”, “sleep”, “mood”, “neurodegenerative diseases”, “immunity”, “phototherapy”, “melatonin”, and “behavior-intervention therapy”. Conference abstracts, meeting posters and unpublished preliminary reports were excluded during literature screening. Additional relevant publications were retrieved by manually checking the reference lists of key included articles.

Because this was a narrative review, no formal risk-of-bias instrument or de novo meta-analysis was applied, no PRISMA flow diagram was constructed, and search-result counts were not retrospectively inferred. The review should therefore not be interpreted as an exhaustive systematic review. Generative AI tools were used to support manuscript organization, language refinement, and preliminary reference cross-checking. The authors independently verified all scientific claims, numerical values, and cited sources against the underlying publications.

## 3. Rhythm

**Figure 1:**
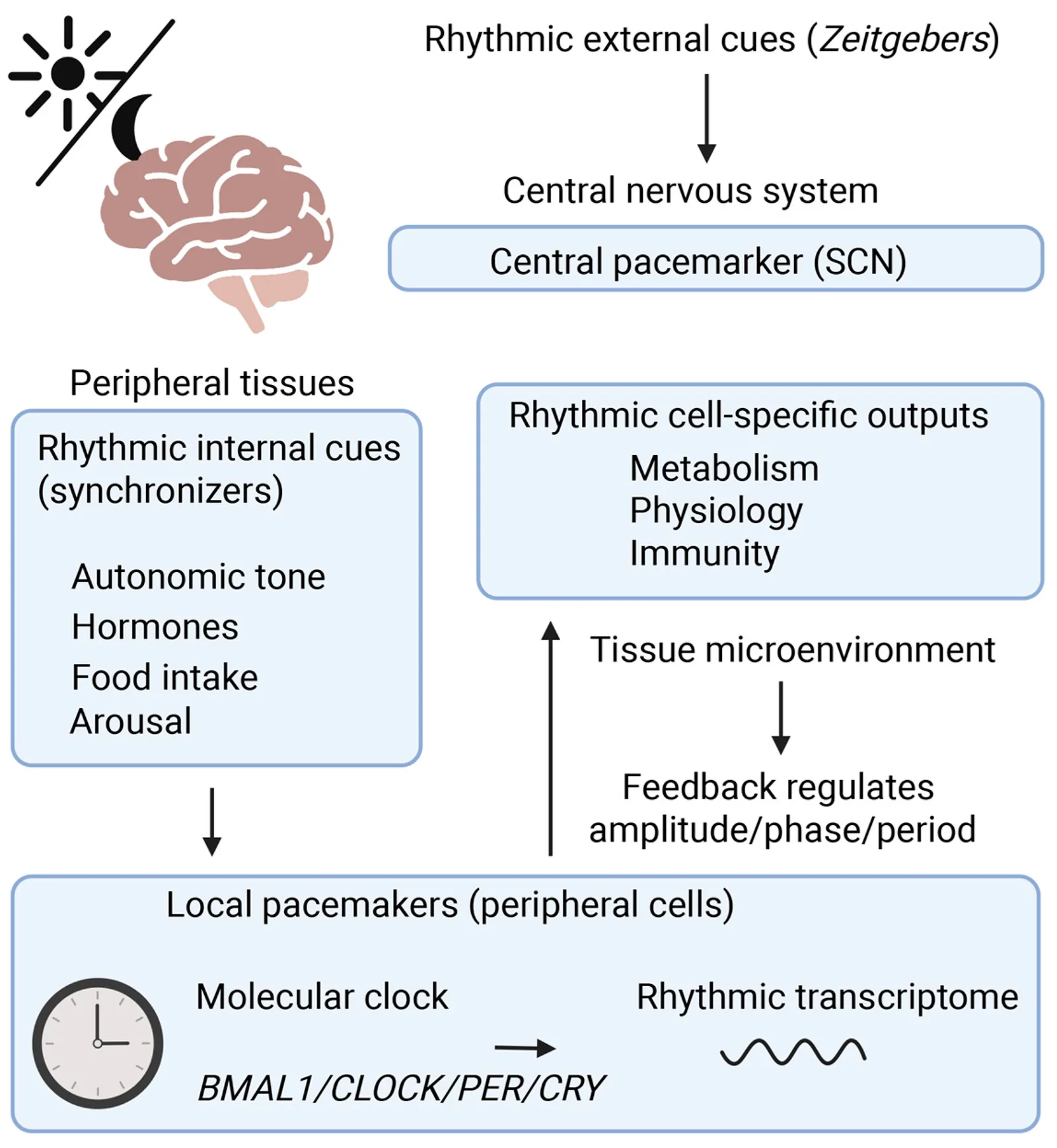
Oscillatory modeling of circadian rhythms. The circadian clock consists of a central pacemaker, the suprachiasmatic nucleus (SCN), which is located in the central nervous system, and local pacemakers in the surrounding tissues. During the daily rhythmic cycle, the clock rhythms of the central pacemaker enter synchronized oscillations of hormone secretion in response to external light, coordinating autonomic activity, appetite and arousal. The peripheral tissue clock is driven by internal synchronizing factors to produce rhythmic output, whereas feedback regulation maintains rhythmic system stability. Created in BioRender. Xu, H. (2026) https://BioRender.com/zdoijls. Accessed on 18 September 2026.

Biological rhythms are recurring changes in physiological and behavioral processes that occur with characteristic periodicity and regularity. Among these, circadian rhythms are approximately 24-h oscillations that coordinate physiological and behavioral processes with the day–night cycle. Circadian rhythms are endogenously generated and can persist under constant conditions, while remaining entrainable to environmental *zeitgebers*, particularly the light–dark (LD) cycle [7,8] (Figure 1). These rhythms contribute to physiological homeostasis by regulating metabolic and cardiovascular functions [9].

Circadian rhythms can be influenced by multiple environmental and behavioral factors. Food intake and exercise can serve as synchronizing cues, whereas light is the principal environmental *zeitgeber* for the central circadian clock [10]. Misalignment between endogenous circadian timing and environmental or behavioral cycles can disrupt sleep–wake timing and contribute to circadian rhythm sleep–wake disorders [11]. Circadian disruption can also have broader physiological consequences, including impaired immune regulation [12]. Therefore, understanding how circadian rhythms are generated, entrained, and disrupted is essential for elucidating the mechanisms through which inappropriate light exposure affects physiological and behavioral functions.

The categorization of rhythm disruptions is largely dependent on the physiological system affected and the mode of presentation. Common categories of rhythm disruptions include sleep–wake rhythm disruptions, heart rate disruptions, and biological rhythm disruptions. Specifically, sleep–wake rhythm disruptions can be subdivided into delayed sleep phase disruptions, advanced sleep phase disruptions, irregular sleep–wake rhythm disruptions, and non-24-h sleep–wake rhythm disruptions [11]. Heart rate disruptions are characterized mainly by arrhythmias, i.e., irregularities in the rhythm of the heartbeat [9]. Biological rhythm disruptions refer mainly to imbalances in the biological clock due to alterations in the external light cycle, leading to a forward or backward shift of the phase.

### 3.1. Molecular Mechanisms of Rhythm

Circadian rhythms enable organisms to coordinate regular changes in physiology and behavior with periodic environmental changes, particularly the day–night cycle. These rhythms are governed by a set of endogenous clock genes, including circadian locomotor output cycles kaput (*Clock*), brain and muscle Arnt-like protein 1 (*Bmal1*), period (*Per*), and cryptochrome (*Cry*). Their rhythmic expression is generated primarily through a transcription–translation feedback loop (TTFL), in which *Clock–Bmal1* heterodimers activate the transcription of *Per* and *Cry* genes, whereas accumulated *PER–Cry* complexes subsequently repress *Clock–Bmal1* activity. Degradation of *Per–Cry* complexes then relieves this inhibition and initiates the next cycle, thereby regulating diverse physiological processes within the organism [13,14,15].

**Figure 2:**
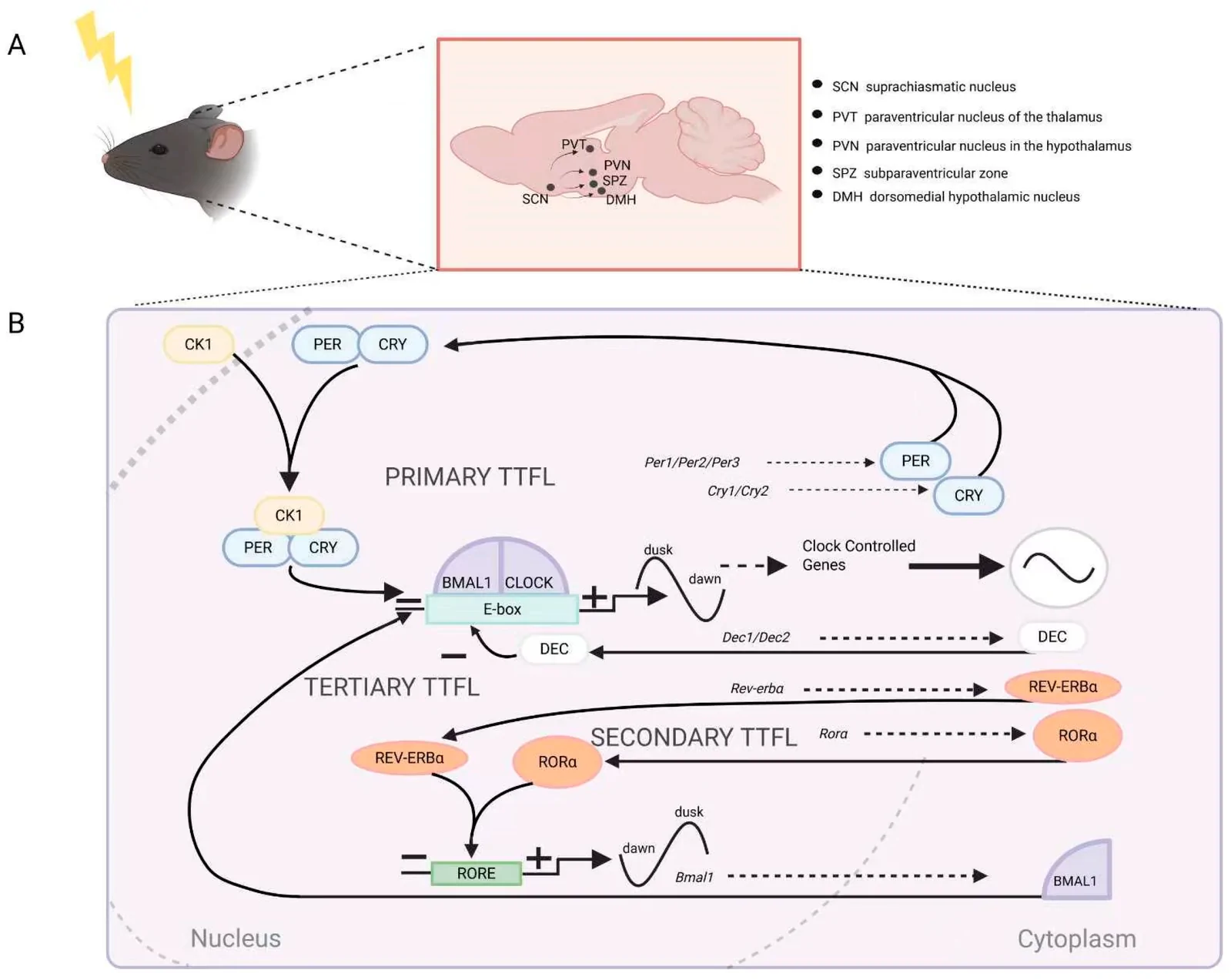
Neuromolecular mechanisms of rhythm. (**A**) The central pacemaker of the central nervous system, the SCN, projects to multiple brain regions, thereby modulating the rhythmicity of different behaviors. (**B**) Rhythmic *Bmal1-Per2* transcription–translation feedback loop. This feedback loop consists of three parts: initiation activation, feedback repression and reactivation. 1. 1. Initiation and activation: BMAL1 heterodimerizes with CLOCK. This dimer binds to the regulatory region of the *Per2* gene and initiates its transcription. The resulting *Per2* mRNA is exported to the cytoplasm and translated into PER2 protein. 2. Feedback inhibition: After excessive accumulation of the PER2 protein in the cytoplasm, it is transported back to the nucleus. In the nucleus, the PER2 protein forms a complex with other related proteins (such as CRY), binding to the corresponding DNA region, inhibiting the transcriptional activity of the *Bmal1-Clock* heterodimer, the transcription of the *Per2* gene, and the production of the PER2 protein. 3. Reactivation: When the PER2 protein is gradually degraded and the inhibitory effect on *Bmal1–Clock* is weakened, the dimer reactivates the transcription of *Per2* and starts the next cycle. Created in BioRender. Xu, H. (2026) https://BioRender.com/zdoijls. Accessed on 4 November 2026.

The regulation of biological clock genes depends not only on endogenous feedback mechanisms but also on environmental cues, particularly light. Light is detected by the retina and transmitted to the suprachiasmatic nucleus (SCN), the central circadian pacemaker, where it entrains and can reset circadian rhythms [16]. Consequently, inappropriately timed light exposure can cause circadian disruption. Circadian disruption has been associated with a broad spectrum of adverse health outcomes, including mood disorders [17], sleep disturbances [18], and metabolic dysfunction [19]. Therefore, maintaining the integrity of these molecular clock mechanisms is important for physiological homeostasis (Figure 2).

### 3.2. BL-Mediated Specific Molecular and Neural Pathways

The biological effects of BL exhibit a dual nature encompassing both physiological regulation and pathological damage. Phase mismatch in BL exposure or light doses exceeding physiological tolerance thresholds transition BL into a pathogenic factor. The mechanisms underlying BL-induced pathological damage unfold primarily through two distinct pathways: one is the non-image-forming (NIF) visual pathway centered on intrinsically photosensitive retinal ganglion cells (ipRGCs) expressing melanopsin, which converts light signals into systemic physiological effects via neural circuits and the circadian system [20]; the other involves localized retinal photochemical damage, in which N-retinylidene-N-retinylethanolamine (A2E)—a lipofuscin fluorophore accumulated in retinal pigment epithelial (RPE) cells—absorbs BL and undergoes photo-oxidation, eliciting oxidative stress and cellular injury [21,22]. These two pathways differ fundamentally in their sites of action and pathological consequences.

At the neural regulation level, the SCN, the principal central pacemaker that receives retinal light input and synchronizes circadian rhythms, has been extensively investigated at the neural-circuit level. As noted above, the SCN integrates retinal light input and conveys circadian timing signals to downstream hypothalamic and extra-hypothalamic regions, thereby broadly regulating the rhythmicity of physiological and behavioral functions, including feeding, hormone secretion, mood-related behaviors, and pain perception. Recent circuit-level studies have shown that the SCN regulates these functions through direct and polysynaptic pathways involving downstream targets such as the dorsomedial hypothalamic nucleus, paraventricular nucleus of the hypothalamus (PVN), subparaventricular zone, and paraventricular nucleus of the thalamus (PVT) [23,24]. Light can influence feeding rhythms in mice through SCN-dependent circuits [24,25]. Separately, nighttime light exposure can induce depressive-like behaviors in mice through a circadian rhythm-gated subcortical pathway involving ipRGCs, the dorsal perihabenular nucleus (dpHb), and the nucleus accumbens [26]. Conversely, activating BL-sensitive M4-type melanopsin-expressing retinal ganglion cells (M4-mRGCs, a subtype of ipRGCs) inhibits CaMKIIα neurons in the lateral habenula (LHb) via a retina–ventral lateral geniculate nucleus/intergeniculate leaflet–LHb (retina–vLGN/IGL–LHb) pathway, serving as an important circuit foundation for the antidepressant effects of light therapy [20,27,28]. In addition, stressors can alter the phase of the SCN circadian clock through specific neuronal inputs [29]. Collectively, by integrating photic and nonphotic inputs and distributing circadian timing signals through distinct downstream circuits, the SCN plays a pivotal role in maintaining organismal rhythmicity. These circuits provide a neuroanatomical basis for examining multisystem disorders associated with inappropriate light exposure and for understanding how light-based interventions may influence disease processes associated with circadian dysfunction.

Distinct from the aforementioned neural pathways, BL can also directly act on RPE cells through A2E-mediated photochemical reactions. As a prominent bis-retinoid component of RPE lipofuscin, A2E markedly enhances the sensitivity of RPE cells to BL phototoxicity [30]. Excitation of A2E by BL generates reactive oxygen species, which subsequently impinge on mitochondrial dynamics and apoptotic signaling cascades, contributing to RPE injury and the pathogenesis of age-related macular degeneration [31,32]. Notably, the formation and accumulation of A2E do not depend strictly on light exposure, as its levels naturally rise with age; thus, A2E-associated phototoxicity is better understood as the joint outcome of endogenous A2E accumulation and BL exposure [33].

Furthermore, nocturnal light exposure can suppress melatonin secretion and disrupt circadian rhythms via the ipRGC–SCN pathway. Chronic light exposure at night and the resulting melatonin suppression have been linked to an increased risk of developing various malignancies, wherein the antioxidant and potential antineoplastic properties of melatonin may represent important underlying mechanisms [34,35,36]. Nevertheless, current evidence pertains predominantly to light at night and circadian disruption, and should not be equated directly with BL being inherently carcinogenic (Table 1).

## 4. Inappropriate Light Exposure Causes Rhythm Disruption

In many cases, inappropriate light exposure triggers circadian disruption. Owing to factors such as artificial lighting, the use of electronic equipment, and shift work, a dislocation between endogenous and exogenous rhythms frequently occurs. For example, athletes competing across time zones suffer circadian misalignment induced by rapid shifts in the light environment, leading to compromised physiological function and athletic performance [42]. Social jet lag is another type of circadian misalignment stemming from inappropriate light exposure linked to irregular daily schedules. Individuals follow the social clock on workdays but revert to their endogenous biological clock on weekends. Such rhythmic shifts increase tobacco, caffeine, and alcohol consumption, which further worsens circadian disruption and creates a vicious cycle [43]. Epidemiological evidence indicates that mismatch between endogenous biological rhythms and external environmental cues (e.g., light exposure and social schedules) elevates the risk of multiple illnesses, including depression, cardiovascular disorders, and metabolic dysfunction [44].

Given the widespread use of modern lighting and the popularity of electronic devices, people are increasingly exposed to inappropriate light environments. Recent animal studies have exposed rats to different doses of BL and white light to assess the effects of spectral components on phototoxicity. The results showed that blue and green spectral components can cause phototoxicity to the retina, whereas the red spectrum possesses protective properties [45]. BL exposure can lead to a reduction in the number of ipRGCs [46]. Given that genetic ablation of ipRGCs has been shown to directly elicit circadian disruption [47], the BL-induced loss of ipRGCs may impair light signal transmission to the SCN and thereby contribute to circadian disruption. This may represent a potential underlying mechanism through which BL exposure mediates circadian dysregulation. Over the past few decades, photic signal transmission function and circadian rhythms may have been adversely affected by the widespread use of LEDs. However, it should be noted that the blue photon flux of LED lighting strongly depends on correlated color temperature (CCT). Warm-white LEDs (approximately 2700 K) produce BL output comparable to or even lower than incandescent lamps, whereas the health risks are primarily associated with cool-white LEDs with CCT > 3000 K, which are widely deployed in commercial and public spaces [48]. There is growing evidence that the use of electronic products has a significant effect on sleep and rhythm. This effect may be related to the shortwave-enriched light emitted by these devices. As early as 2015, a study compared the biological effects of using e-books and paper books a few hours before bedtime. Participants who used e-books were found to take longer to fall asleep, report reduced sleepiness at night, and show suppressed melatonin production, thereby delaying the phase of their biological clock [49]. This suggests that exposure to electronic light at night can have a significant negative impact on sleep.

In conclusion, these environmental factors have important impacts on rhythm disruptions and physical health. An in-depth understanding of the mechanisms and hazards of light-induced rhythm disruptions is important for raising public awareness regarding unhealthy light exposure and promoting appropriate preventive and corrective measures.

## 5. Light-Induced Circadian Disruption and Associated Diseases

**Figure 3:**
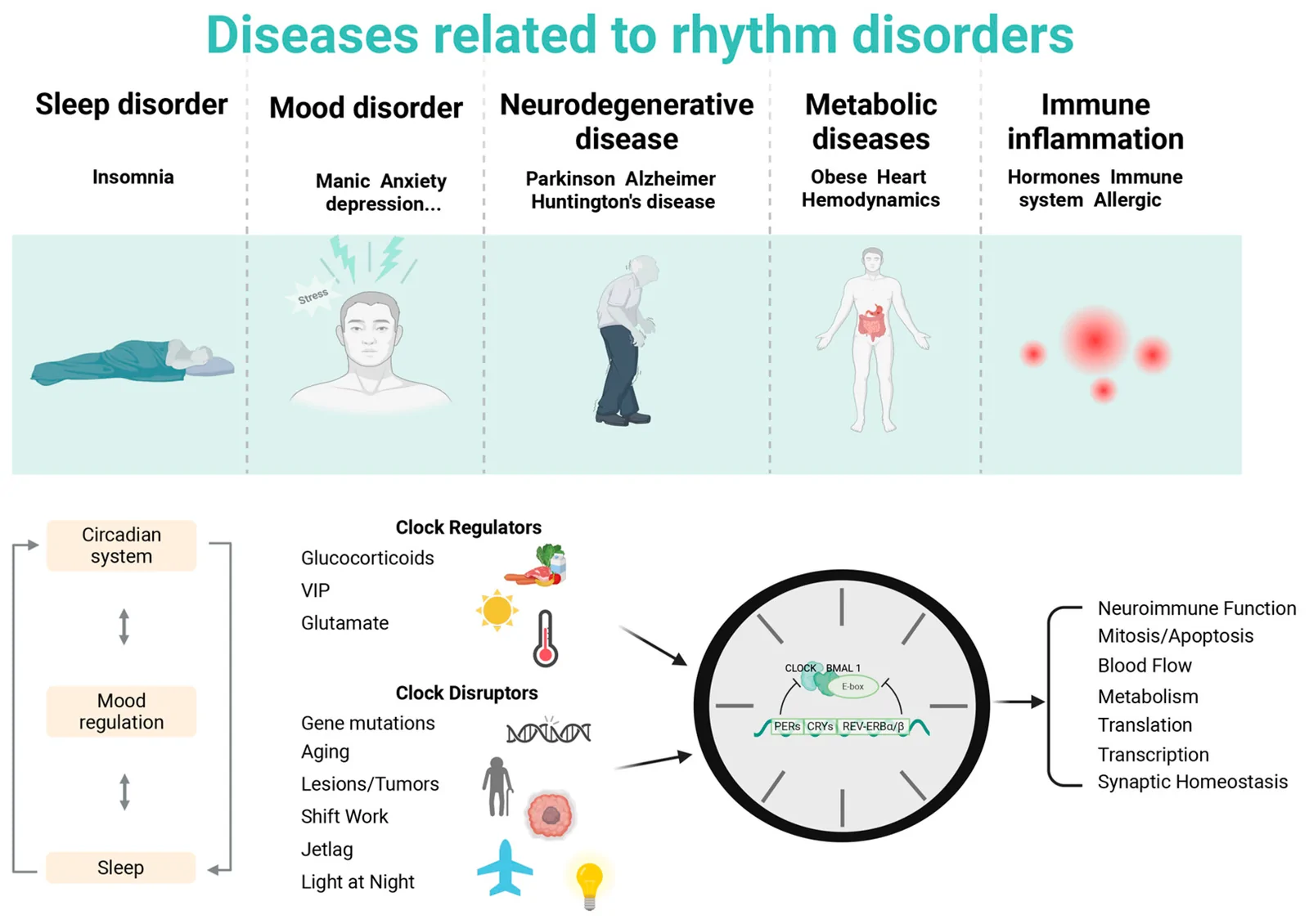
Rhythm-related diseases. Rhythm disruptions are directly linked to sleep disruptions, mood disruptions, neurodegenerative disruptions, and metabolic and immune disruptions. Diseases interact with each other, such as rhythm disruptions leading to sleep–wake dysregulation, which in turn affects mood. Biorhythmic regulation is influenced by environmental factors, and several neurotransmitter hormones play a coordinated role, ultimately affecting neural function, cellular function, metabolism, and synaptic connectivity. Created in BioRender. Xu, H. (2026) https://BioRender.com/zdoijls. Accessed on 18 September 2026.

Circadian disruption is tightly linked to the pathogenesis and progression of diverse human disorders across neurological, metabolic, and cardiovascular systems. Inappropriate light exposure disrupts endogenous clock signaling and serves as a modifiable environmental risk factor for these rhythm-associated diseases (Figure 3).

### 5.1. Sleep Disorders

Circadian sleep disorders are frequently attributed to altered melatonin secretion induced by inappropriate light exposure. Abnormal light signals may suppress melatonin release, disrupt endogenous circadian timing, and thereby trigger sleep disturbances. Melatonin is an important hormone that regulates circadian rhythms and sleep–wakefulness; it is secreted primarily by the pineal gland, but is also present in the retina and gastrointestinal tract. Previous studies have demonstrated that dim light at night suppresses melatonin secretion (7:30 PM–10:00 PM) [50]; this finding has drawn widespread attention to light-induced rhythm disruptions. Further research revealed that artificially increased BL competed with natural light when the workplace light color temperature was set between 4000 Kelvin (K) and 8000 K. When working in warm light (4000 K), subjects aligned with natural dawn, whereas subjects exposed to BL entered office hours prematurely [51].

The invention of LEDs rapidly led to the development of high-efficiency lighting installations as well as LED screen devices. Unlike conventional lighting, most cool-white LED lamps emit wavelengths falling within the BL range, which is close to the peak sensitivity of melanopsin. Research has shown that exposure to BL before bedtime at night can inhibit melatonin secretion [52], increase alertness [53], interfere with sleep [54,55] and reduce cognitive ability [56,57]. In addition, individuals with delayed sleep schedules who are accustomed to late bedtimes display heightened sensitivity to BL [58,59]. Therefore, the intensity and spectral wavelength of indoor and outdoor lighting represent particularly critical factors essential for human health and survival. With the widespread adoption of smartphones, public awareness regarding the adverse effects of prolonged mobile phone use has increased. A study analyzing health changes in users exposed to smartphone BL clearly indicated that altering the circadian illuminance spectral distribution of mobile devices significantly reduces melatonin secretion, thereby impacting human health and circadian rhythms [60]. It is suggested that optimizing the spectral power distribution and managing screen light intensity serve as more practical interventions against screen BL than abruptly shutting down devices. In summary, rational management of the light environment is crucial for preserving melatonin secretion and promoting overall physiological health.

### 5.2. Mood Disturbances

The BL emitted by electronic devices at night increases the likelihood that individuals become susceptible to sleep disruptions, depression, and anxiety associated with mobile phone use [3,61]. An early study examined the correlations between mobile phone addiction, sleep, anxiety, and depression in 319 college students (203 females, 116 males; mean age 20.5 ± 2.45 years). The results revealed that women scored higher on the mobile phone addiction scale than men did, and prolonged exposure to mobile phone screens aggravated anxiety, depression, and daytime dysfunction [62]. However, this does not imply that complete BL filtering exerts solely beneficial effects on emotional regulation. A 2020 study confirmed that filtering out the BL component from normal ambient light induced depression-like behaviors in gerbils [63], accompanied by melatonin secretion changes, HPA axis abnormalities, and 5-hydroxytryptamine reduction. Furthermore, c-Fos expression in the arcuate nucleus of the hypothalamus was significantly increased in the monochromatic BL group, whereas the expression of phosphorylated extracellular signal-regulated kinase (p-ERK) was decreased in the PVN [64], suggesting that specific brain regions participate in the BL-induced emotional response. By monitoring day and night sleep rhythms, studies have shown that circadian rhythm disruptions often precede emotional symptoms, highlighting the potential impact of circadian management on mood disorders. For example, sleep or light interventions can restore circadian rhythms, thereby effectively managing emotional disturbances.

Accumulating evidence demonstrates that gut microbiota, beyond regulating host digestion, metabolism, and immune function, can modulate emotional and mental states via the microbiota–gut–brain (MGB) axis [65,66]. Gastrointestinal microbiota are closely linked to host sleep and circadian rhythms. Acute 72-h sleep deprivation disrupts colonic circadian rhythms and triggers gut dysbiosis, which reduces short-chain fatty acid production, activates intestinal inflammation, and impairs intestinal barrier integrity [67]. Inflammatory signals are bidirectionally transmitted along the MGB axis, further triggering microglial activation and the upregulation of chemokines. Existing studies indicate that sleep deprivation induces gut dysbiosis and excessive inflammatory responses, ultimately exacerbating emotional disturbances and cognitive impairment [68]. Notably, aberrant light exposure (e.g., nighttime BL), a critical environmental modulator of circadian rhythms, can directly cause circadian disruption, subsequently leading to sleep disturbances, gut dysbiosis, and compromised immune function. Accordingly, light-induced circadian rhythm disruptions give rise to sleep disruption, mood disorders, and immune abnormalities that interplay with each other, forming a complex pathological vicious cycle.

### 5.3. Neurodegenerative Disease

**Figure 4:**
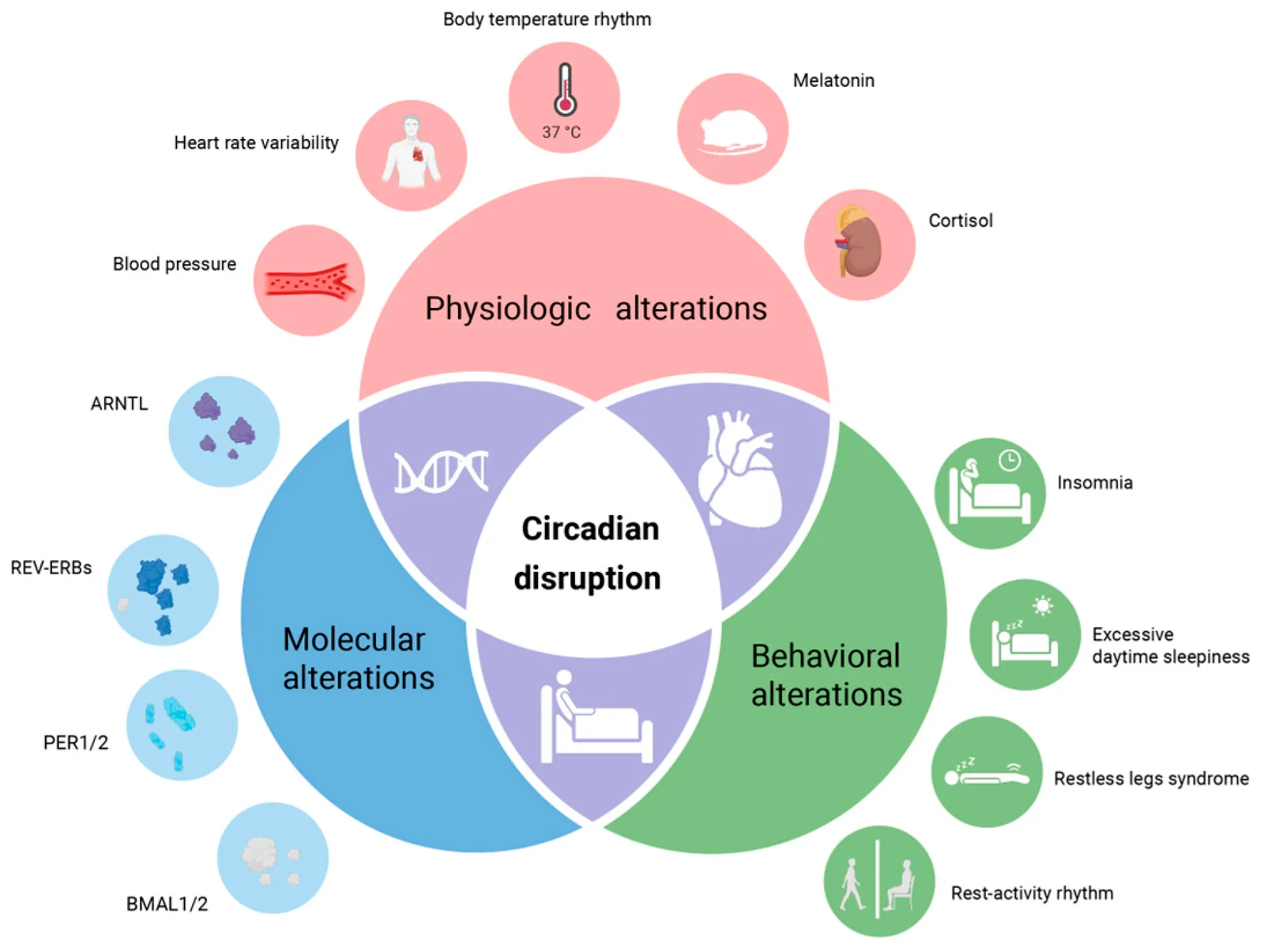
Rhythm disruptions in neurodegenerative diseases. Circadian rhythm disruptions in neurodegeneration can be divided into three categories: behavioral, physiological, and molecular alterations. Rhythms are affected by both sleep disruption and neurodegeneration. Created in BioRender. Xu, H. (2026) https://BioRender.com/zdoijls. Accessed on 18 September 2026.

In recent years, research on neurodegenerative diseases such as Alzheimer’s disease (AD) and Parkinson’s disease (PD) has expanded significantly. However, exploring the pathogenesis of neurodegenerative disorders from the perspective of light-regulated circadian rhythm disruption remains a relatively emerging field. Abnormal exposure to or disruption of light—a key environmental factor regulating the biological clock—may directly or indirectly influence the pathogenesis and progression of neurodegenerative disorders. Research suggests that circadian disruption induced by abnormal light environments is implicated in AD [69,70], PD [71], HD, amyotrophic lateral sclerosis, and other neurological disorders (Figure 4).

#### 5.3.1. Parkinson’s Disease

As illustrated in Figure 4, neurodegenerative diseases are frequently closely associated with circadian rhythm disruptions. Studies have demonstrated bidirectional interactions between PD and disturbances in the circadian molecular clock. Sleep disturbances associated with early-stage PD may stem from intrinsic molecular clock abnormalities of physiological rhythms, while pathological alterations induced by the disease itself can further disrupt circadian-related molecular and hormonal homeostasis, with the two conditions interacting and exacerbating one another. Further studies, such as an 11-year follow-up survey of 2930 men published in 2020, have shown that the risk of PD increases with decreasing circadian rhythmicity [72]. Sleep disturbance is one of the most prevalent non-motor symptoms of PD [73], and the vast majority of patients subjectively report impaired sleep quality. Studies have demonstrated that patients with PD exhibit prolonged sleep latency, reduced sleep efficiency, and a decreased proportion of rapid eye movement sleep, accompanied by elevated serum cortisol levels, reduced melatonin levels, and aberrant *BMAL1* gene expression [74]. In addition, light exposure can induce changes in clock gene expression, and variations in clock genes can affect mitochondrial dynamics [75] as well as the rhythmic synthesis and secretion of endocrine hormones (such as melatonin), thereby contributing to the pathogenesis of PD. According to a 2023 study, circadian pacemakers function as presynaptic neurons to dopaminergic neurons, and null mutations in the Drosophila PER gene exacerbate the age-dependent loss of dopaminergic neurons, interacting with transient oxidative stress to cause premature death in animals [76]. These findings suggest that light-regulated circadian disruptions may promote dopaminergic neuron degeneration, and the loss of nigrostriatal dopaminergic neurons represents a hallmark feature of PD. Evidence indicates that age-related ocular and neurological conditions are indeed directly associated with light-induced circadian rhythm disruptions [77,78]. For example, cataracts and glaucoma reduce light input to the SCN and further aggravate the manifestations of neurodegenerative disorders. Weiwei Liu et al. provided a detailed review of proteomic techniques used to analyze pathways associated with the pathogenesis of neurodegenerative disorders. These findings provide new avenues for precisely elucidating the mechanisms of light-mediated circadian rhythm disruption in neurodegenerative disorder pathology.

#### 5.3.2. Alzheimer’s Disease

In addition, aging is also associated with light-induced circadian rhythm disruptions, suggesting that light-induced circadian rhythm disruptions may serve as a biomarker for AD and help us understand disease pathogenesis. A study revealed that AD is associated with temporal differences in the expression of physiological markers of circadian rhythms, and that multiple factors, particularly light-induced circadian disruption, lead to changes in the expression of rhythmic markers that may exacerbate AD progression. Researchers analyzed transcriptomic and epigenomic data from patients with AD and found that their circadian rhythm was phase-advanced by nearly 1.5 h compared to the control group, whereas their seasonal rhythm was delayed by half a month [79]. These brain-wide, site-specific, and interrelated circadian and seasonal rhythm signatures show that light-regulated rhythm changes represent an important biological feature of the brain and may constitute contributing factors. Musiek ES et al. analyzed daily rest–activity rhythm actigraphy data from 189 participants and reported that AD was associated with disruptions in resting-activity rhythms [80], and demonstrated that SCN astrocyte molecules are sufficient to drive the daily cycle of neuronal activity and behavior. A previous study revealed that rhythm disruption can accelerate the activation of astrocytes and other pathological brain lesions in an AD mouse model, indicating that light-induced circadian disruption can act between astrocytes and neurons to accelerate the onset and progression of AD. Although most of these studies were observational and utilized different quantitative methods, they consistently suggest that light-induced circadian disruption causes alterations in gene expression, functional phenotypes, and functional variants that may have important implications for AD.

### 5.4. Metabolic Diseases

The circadian system consists of the central clock, which is hosted by the SCN located in the hypothalamus, and peripheral tissue clocks. Its core function is regulated by light signals. The system plays a key role in coordinating a variety of daily functions, including metabolism, hormone secretion, the cell cycle, and locomotor activity. The central clock regulates food intake, energy consumption, and systemic insulin sensitivity through light signals, and these effects are fine-tuned by peripheral clocks. However, inappropriate light exposure may lead to circadian disruption in the body, increasing the risk of metabolic diseases [7,81,82].

Studies have shown that light-induced sleep disruption increases the body’s demand for carbohydrates and fats, leading to obesity and metabolic syndrome in the long run [83]. In animal studies, a cafeteria diet following social jet lag can induce overeating and weight gain while producing adverse metabolic profiles, including hyperinsulinemia and elevated diabetes risk, in rats [84]. Patricia Wong et al. conducted a study on 447 healthy adults (53% female, average age 42 years, 83% Caucasian) to explore the relationships among chronotype, social jet lag, and cardiometabolic risk via light regulation. The study assessed hemodynamics, metabolic variables, and depressive symptoms, and recorded behaviors related to smoking, alcohol consumption, physical activity, and food intake. Stratified regression analysis revealed that social jet lag disrupts the internal rhythm of physiological processes such as glucose metabolism, body temperature, and blood pressure regulation, thus impairing normal cardiometabolic function [85]. Another study revealed that blood pressure and serum angiotensin II levels were significantly increased in mice when *CLOCK*—a core clock gene affected by light—was knocked out; furthermore, light-induced circadian disruption driven by chronic kidney disease upregulates G protein-coupled receptor 68 expression in monocytes, which further exacerbates cardiac inflammation and fibrosis [86]. These findings indicate that light-induced circadian disruption drives adipose accumulation and hormonal dysregulation through multiple pathways, thereby promoting the development of obesity and metabolic syndrome. However, it should be noted that these conclusions are derived predominantly from animal models; species differences between rodents and humans, particularly in circadian activity patterns and metabolic regulation, limit direct extrapolation.

In the peripheral rhythm system, studies have revealed dysregulation of photoregulated clock genes and cell cycle regulators (such as *cyclin D1*, *c-Myc*, *Wee1*, and *p27*) in the parathyroid glands and aortas of uremic rats, resulting in dysregulation of parathyroid cell proliferation, which in turn affects the body’s metabolic function [87]. Further exploration of the regulatory mechanisms governing cell division and metabolism revealed that the disruption of rhythms by light signaling alterations (e.g., shift work) is directly related to cancer progression and poor prognosis; thus, circadian homeostasis is recognized as having a tumor-suppressive role. Specifically, using a genetically engineered mouse model of lung adenocarcinoma, one study demonstrated that loss of the core clock genes *Per2* and *Bmal1* resulted in elevated c-Myc expression, enhanced cell proliferation, and metabolic dysregulation, thereby facilitating lung tumor growth and progression [88]. Consistent with observations from animal models of uremia described above, preclinical mouse systems can only recapitulate partial molecular alterations seen in human malignancy. Accordingly, findings derived from such animal experiments cannot be directly equated with the pathological state of genuine human diseases.

### 5.5. Immune Inflammation

The adrenergic system is dependent on catecholamine neurotransmitters such as epinephrine and norepinephrine. The synthesis of these hormones may be regulated by a light-entrained central clock. Thus, we can observe rhythmic oscillations in catecholamine levels in the blood and peripheral tissues. This oscillation in catecholamine levels may affect multiple adrenergic receptor cellular targets, including immune system cells [89]. In addition, studies have shown that light-regulated melatonin metabolism is altered in allergic patients. As a light-regulated circadian hormone, melatonin can coordinate not only the differentiation and function of immune cells, but also the severity of many allergic diseases, which is closely related to the circadian function of the immune system. Therefore, as a rhythm-regulating hormone, melatonin may indirectly modulate immune function via the light-entrained biological clock and is associated with food allergies [90]. Godinho-Silva, C et al. reported that group 3 innate lymphoid cells, as major regulators of inflammation, infection, microbial composition, and metabolism, are regulated not only by light but also by circadian rhythms [91]. In summary, light-induced circadian disruption can exert negative effects on immune function, which are characterized mainly by allergies, inflammation, and infection. Moreover, maintaining rhythm homeostasis through the coordination of a normal light environment holds clinical application potential for enhancing host immunity.

## 6. Current Controversies Regarding Methodological Determinants of BL-Induced Circadian Effects

Although the circadian effects of BL have been extensively characterized, considerable inconsistencies remain regarding the magnitude of biological responses reported across experimental studies. These discrepancies are primarily attributable to differences in light exposure parameters, including spectral composition, intensity, exposure timing, and duration, rather than representing fundamentally contradictory biological observations. Therefore, BL should not be interpreted as a uniform stimulus; instead, its circadian effects depend on the integrated characteristics of the photic environment and its relationship with the endogenous circadian phase.

The contribution of wavelength has been established through controlled monochromatic-light experiments, demonstrating that shorter wavelengths within the blue spectrum generally exert greater circadian potency than longer wavelengths under comparable photon exposure conditions. Exposure to approximately 460 nm light produces stronger melatonin suppression and circadian phase-shifting effects than 555 nm light, supporting the predominant contribution of melanopsin-containing ipRGCs to non-image-forming photic responses [52]. However, the relationship between wavelength and circadian sensitivity is not completely explained by melanopsin activation alone. Subsequent dose–response studies demonstrated that spectral sensitivity changes with irradiance and exposure duration, suggesting that cone photoreceptor inputs and temporal adaptation processes also contribute to human circadian phototransduction [92]. More recent findings using calibrated spectral manipulation further indicate that changes in chromatic composition at matched melanopsin stimulation do not always produce substantially different circadian outcomes, emphasizing that wavelength effects should be interpreted within the context of the overall photoreceptor activation profile rather than wavelength alone [93].

Light intensity represents another major determinant explaining variability among studies. Laboratory investigations using high irradiance conditions typically demonstrate robust melatonin suppression and phase resetting, whereas studies using realistic indoor lighting or electronic devices often report smaller physiological changes. This difference is particularly evident when comparing controlled monochromatic protocols with everyday screen exposure. For example, evening exposure to LED-based electronic devices can alter melatonin secretion and circadian timing despite relatively moderate real-world illumination levels [49,56], whereas classical laboratory protocols frequently apply substantially higher photon densities to define circadian dose–response relationships. Importantly, illuminance (lux) alone cannot fully describe circadian potency because visual sensitivity-weighted measurements do not accurately represent melanopsin-mediated responses. Consequently, comparisons between studies require consideration of spectral irradiance, photon density, and melanopic stimulation rather than relying exclusively on conventional photometric units.

The timing of exposure provides a critical explanation for apparently opposing effects of BL. The same light stimulus may induce circadian disruption or facilitate circadian realignment depending on when it is delivered relative to the internal clock phase. Evening and nighttime exposure, particularly around the onset of melatonin secretion, produces strong melatonin suppression and phase delays, whereas appropriately timed morning exposure can promote phase advances and improve circadian alignment. This temporal dependence explains why blue-enriched light is associated with impaired sleep when encountered before bedtime but may provide therapeutic benefits when administered during the morning or at a biologically appropriate phase [55,94,95]. The circadian response therefore depends not only on the external clock time of exposure but also on individual differences in chronotype and endogenous circadian timing.

Exposure duration further contributes to heterogeneity among experimental findings. Earlier studies frequently employed prolonged continuous illumination, such as approximately 6.5-h nighttime exposures, to characterize maximal circadian responses [52]. However, subsequent investigations demonstrated that circadian responses do not increase linearly with exposure duration. Different exposure lengths can produce distinct levels of melatonin suppression and phase shifting, indicating that the relationship between duration and biological response follows a nonlinear pattern rather than a simple cumulative-dose model. In addition, intermittent light exposure paradigms have shown that appropriately timed brief light pulses can induce measurable circadian effects, further challenging the assumption that continuous illumination is required for effective circadian modulation [92].

Taken together, current evidence suggests that the apparent contradictions in the BL literature mainly arise from differences in experimental design and exposure conditions. Wavelength determines the spectral efficiency of photoreceptor activation, intensity defines the magnitude of retinal stimulation, timing determines the direction of circadian adjustment, and duration influences the cumulative and temporal characteristics of photic input. Future investigations should therefore adopt standardized reporting frameworks incorporating spectral distribution, irradiance, melanopic stimulation, circadian phase, and exposure duration to enable meaningful comparison across experimental and clinical studies.

## 7. Therapeutic Strategies Targeting BL–Circadian Interactions

### 7.1. Interventions for Circadian Disruption Induced by Inappropriate BL Exposure

Clinical treatment methods for circadian disruptions caused by multiple factors mainly include exogenous melatonin supplementation, lifestyle modifications (such as diet and exercise), and light therapy strategies. Current research demonstrates that these approaches are effective in alleviating complications such as sleep disturbances, mood disorders, and even neurodegenerative diseases induced by circadian disruption.

In summary, light exposure not only serves as a non-pharmacological strategy for improving sleep and restoring biological rhythms, but also exerts positive effects on systemic inflammation and lipid metabolism in clinical practice [96], thereby expanding the application prospects and innovative potential of light-based therapies in the medical field.

#### 7.1.1. Drug Treatment

Melatonin is synthesized in several tissues, including the pineal gland and retina, and its production is directly modulated by photic input. Previous studies have shown that melatonin influences the expression of clock genes in the mouse retina, while disruption of melatonin receptor signaling can alter normal circadian rhythmicity [97]. Melatonin receptors are also expressed in the SCN, the central circadian pacemaker, where melatonin signaling contributes to the regulation of circadian timing and the sleep–wake cycle.

Pharmacokinetic studies of exogenous melatonin supplementation have shown that plasma melatonin concentrations peak approximately 41 min after administration, consistent with a typical absorption window of 30–60 min. These findings suggest that administration approximately 40 min before bedtime may be appropriate for promoting sleep onset [98]. Moreover, exogenous melatonin appears to have a low propensity to induce clinically meaningful tolerance or dependence, supporting its potential as an effective therapeutic option with a favorable safety profile [99].

The chronobiotic effects of melatonin have been widely applied to the management of sleep disturbances associated with circadian disruption caused by inappropriate light exposure, including BL exposure [100]. Such applications include jet lag, shift-work disorder, and delayed sleep–wake phase disorder. In a double-blind, randomized, placebo-controlled trial involving 331 patients with insomnia, exogenous melatonin supplementation improved sleep quality and morning alertness, while the reduction in sleep latency was reported to be comparable to that achieved with hypnotic medication. After three weeks of treatment with 2 mg melatonin daily, participants in the treatment group showed improvements in sleep outcomes relative to those receiving placebo [101]. In sleep-deprived participants, administration of 1.8 mg melatonin produced a beneficial effect on sleep duration from the first day of treatment. By comparison, treatment with 6 mg melatonin for three consecutive days was associated with improvements in attention and cognitive performance, together with increases in daily melatonin levels [102]. No evidence of rebound insomnia or adverse phase-response effects was observed following treatment discontinuation, and the intervention was generally well tolerated [103].

As discussed in Section 5.2, complete elimination of BL may provide little benefit and may, under some circumstances, adversely affect mood. A similar principle appears to apply to melatonin-based interventions: several studies have suggested that combining appropriately timed melatonin administration with appropriately timed light exposure can produce greater circadian phase shifts than either intervention alone, although treatment protocols and timing vary considerably across studies. For example, one randomized controlled trial reported that afternoon melatonin administration alone produced a 0.72-h phase advance, whereas morning light exposure alone produced a 0.31-h phase advance. When the two interventions were combined, the phase advance reached 1.04 h (*p* < 0.0002) [104].

Nevertheless, the available evidence has several important limitations. Most relevant randomized controlled trials have enrolled either healthy volunteers or individuals with specific comorbid conditions, and sample sizes have generally been modest. Considerable heterogeneity also exists across studies with respect to light intensity, spectral composition, melatonin dose, and the timing of both light exposure and drug administration, limiting direct comparisons between studies.

Furthermore, the benefits of combined melatonin and light-based interventions have been demonstrated primarily for circadian phase advancement, whereas evidence regarding phase delays and broader clinical outcomes remains limited. No clear consensus has yet been established regarding the optimal timing window, dosing strategy, or target population. Further well-designed clinical studies are therefore required before these combined interventions can be translated into standardized clinical practice.

#### 7.1.2. Behavioral and Lifestyle Interventions

Light-induced circadian disruption can induce sleep disturbances, emotional abnormalities, and neurological comorbidities, which require safe and effective intervention strategies. Although these phenotypes often appear together following circadian misalignment, they are mediated by partially distinct biological pathways, highlighting the need for safe and effective intervention strategies. Among existing therapeutic regimens, behavioral intervention serves as a low-risk and highly feasible approach, with physical exercise and dietary regulation exhibiting prominent modulating effects on circadian dysfunction. Combined with conventional phototherapy and pharmacological treatments, these interventions can comprehensively alleviate symptoms related to circadian disruption and improve patient prognosis.

Behavioral intervention therapy is one of the safest modalities for the treatment of light-induced circadian disruption. As a core behavioral measure, physical exercise plays a critical role in circadian rhythm regulation. A survey investigating adolescent sleep conditions under different exercise regimens indicated that morning light exposure and morning exercise benefit sleep promotion, whereas nighttime exercise before sleep should be avoided [105]. Animal studies further demonstrated that physical exercise can remodel impaired circadian systems and relieve depression- and anxiety-like behaviors in gerbil models with light-induced circadian disruption, confirming the capacity of physical activity to regulate circadian disorders and related comorbidities. In addition, in R6/2 transgenic mouse models of Huntington’s disease, bright light therapy and voluntary exercise delayed the progressive disintegration of rest-activity rhythms and improved behavioral synchronization to the light–dark cycle, with the greatest benefits observed when bright light therapy was combined with restricted periods of voluntary exercise [106]. Collectively, these findings support appropriately timed physical activity as a potential behavioral strategy for improving disrupted circadian rhythmicity across different experimental contexts.

In addition to exercise, dietary modification has been investigated as a potential behavioral strategy for addressing adverse outcomes associated with circadian disruption. A literature review of five studies involving a total of 670 night-shift workers evaluated longer-term nutritional programs and shorter-term modifications of meal composition, type, and timing. However, the findings were heterogeneous. In the two studies that assessed sleepiness, dietary modification did not produce significant improvements, and no significant improvement in alertness was observed in the relevant comparison of high-protein or high-carbohydrate diets with a regular control diet [107]. Thus, current evidence does not support a consistent beneficial effect of dietary interventions on sleepiness or alertness in night-shift workers.

Time-restricted eating has also been proposed as a potential intervention for individuals with circadian and sleep disturbances. A randomized controlled trial protocol is currently designed to compare time-restricted eating with a Mediterranean diet in individuals with bipolar disorder and co-occurring sleep or circadian rhythm disturbances. The protocol reports preliminary, uncontrolled survey findings in individuals who had previously attempted time-restricted eating, some of whom self-reported improvements in sleep timing, ability to sleep through the night, mood, and quality of life. However, these preliminary observations did not include a Mediterranean-diet comparison and therefore do not establish the superiority of time-restricted eating [108]. Overall, dietary timing and composition remain promising targets for circadian-related interventions, but their effects on sleep, alertness, mood, and circadian function require further confirmation in adequately controlled clinical trials.

In summary, current treatments for circadian disruption include phototherapy, medication, and behavioral interventions, boasting a favorable safety profile. These interventions can effectively improve sleep and mood disorders caused by circadian dysregulation and relieve additional symptoms in patients with concurrent neurodegenerative diseases. Therefore, light modulation-based therapeutic strategies for circadian disruption possess promising clinical application potential. Such multimodal intervention approaches may gradually translate into clinical practice and offer more holistic, individualized management plans for patients with circadian rhythm disorders, while we recognize that definitive clinical benefits remain to be fully confirmed.

### 7.2. Therapeutic Applications of Controlled BL Exposure

BL can simultaneously trigger a set of interrelated physiological responses including melatonin suppression, modulation of circadian rhythms, acute changes in alertness, and mood regulation. While these phenotypes often co-occur following blue-light exposure and share upstream photodetection via ipRGCs, they are mechanistically distinct, relying on divergent downstream neural circuits. Modern LED light sources generally contain BL components. Compared with 4000 K white light, LED-derived BL exerts a stronger inhibitory effect on melatonin synthesis [109]. In addition, monochromatic BL is more effective at increasing human alertness than long-wavelength light [110]. Based on these optical properties, targeted BL exposure and BL-blocking interventions exhibit broad application potential in sleep regulation, emotional disorder management, and adjuvant therapy for neurological diseases.

In terms of circadian sleep regulation, clinical and environmental studies have demonstrated that strategic exposure to short-wavelength light at night, or the use of BL-blocking glasses during the day [111], can modulate sleep parameters. Research conducted in polar winter environments confirms that BL facilitates earlier sleep onset relative to standard white light [54,55]. Clinical studies in special populations further validate the sleep-improving effects of BL. Patients with mild traumatic brain injury (mTBI) receiving 30-min daily BL pulse treatment for six weeks showed circadian phase advance and alleviated daytime sleepiness [94,112,113].

Regarding cognitive function, BL intervention exerts beneficial effects on cognitive and executive functions across different populations. For pathological populations, BL therapy improves executive performance in patients with mTBI [94,113] and enhances cognitive function in patients with AD [95]. In healthy, normally rested adults, a 30-min exposure to blue-wavelength light has been associated with faster responses during a subsequent working-memory task and with improved retention of verbal material when administered during memory consolidation [114,115]. Under acute hypoxic conditions, blue-light exposure has also been associated with favorable trends in some cognitive measures, although the overall evidence remains inconclusive [116].

For mood and affective disorders, preclinical animal experiments have verified that BL intervention yields potential antidepressant effects in mouse models [112]. In clinical treatment of affective disorders, low-intensity BL (750 lux) achieves comparable efficacy to high-intensity white light (10,000 lux) in relieving or reversing symptoms of seasonal affective disorder [117,118].

BL therapy also shows promising prospects as an adjuvant intervention for HD. Animal experiments have demonstrated that three months of continuous BL irradiation ameliorates motor performance in HD model mice, offering a novel research direction for adjuvant clinical treatment of HD [6]. To date, no controlled clinical studies have investigated the efficacy of phototherapy in patients with HD. In contrast, available clinical evidence indicates that phototherapy applied either alone or combined with melatonin supplementation yields beneficial effects in patients with AD and PD [72,73,74]. Although rodent model studies provide fundamental biological insights into the genetic mechanisms underlying HD pathogenesis, these basic experimental findings are chiefly valuable for elucidating molecular and genetic pathways. Notably, animal-derived outcomes cannot be directly extrapolated to human clinical settings except for investigations related to gene therapy. Accordingly, ambient light regulation and phototherapeutic intervention may serve as adjunctive management strategies for HD and other neurodegenerative disorders, whereas rigorous verification is still required for the clinical translation of preclinical observations on circadian modulation.

The regulatory effects of BL on humans exhibit prominent interethnic and individual variability, which is primarily associated with ocular pigmentation characteristics. A 2007 study confirmed that differences in ocular pigmentation between Caucasian and Asian populations directly modify the melatonin-suppressive response to light [119]. Specifically, individuals with blue eyes display higher summertime cone sensitivity than those with dark eyes [120]. Populations with dark eyes are more susceptible to depression, fatigue, and delayed sleep–wake phase disorder.

In summary, BL therapy possesses important application value in multiple medical fields, including sleep regulation, cognitive enhancement, mood intervention, and adjuvant treatment of neurodegenerative diseases. Nevertheless, its effects vary individually due to ethnicity, personal physiological traits, and health status (Table 2).

## 8. Conclusions and Future Outlook

Rhythmicity is one of the fundamental features that emerged during biological evolution and is involved in the regulation of a wide range of physiological processes, including sleep, endocrine function, emotion, and immunity. Among the various biological rhythms, circadian rhythm disruption is closely associated with human health and has become an important area of research. However, previous studies have mainly focused on its molecular mechanisms and behavioral periodicity, whereas relatively limited attention has been paid to how circadian disruption affects behavior and whether its long-term cumulative effects contribute to disease onset and progression. With advances in molecular biology and neural circuit research techniques, light, as an important environmental *zeitgeber*, has attracted increasing attention. Whether excessive light exposure in daily life, particularly BL exposure at night, affects health by inducing circadian disruption, and the underlying mechanisms involved, remain to be systematically elucidated. Clarifying these issues may provide a new theoretical basis and potential targets for the prevention and intervention of related diseases.

Existing animal studies have shown that abnormal light exposure can disrupt circadian homeostasis and is accompanied by a range of behavioral, physiological, and biochemical alterations. Nevertheless, substantial limitations remain in this field, hindering the translation of findings from basic research into clinical applications. At the level of animal models, most studies have primarily focused on the effects of light exposure on animal behavior and physiological or biochemical parameters, whereas the mediating or regulatory role of circadian disruption in light-related injury has not been fully established. The molecular and circuit mechanisms underlying the adverse effects of light exposure also remain incompletely understood, and it is difficult to distinguish the direct effects of light from indirect effects secondary to circadian disruption. Therefore, the key pathways involved and their causal relationships require further clarification. In addition, most current animal studies employ relatively simple light-exposure paradigms, which cannot fully recapitulate the more complex lighting environments encountered in real-world human life.

Clinical studies suggest that abnormal light exposure is associated with sleep disturbances, circadian disruption, and mood abnormalities, while sleep disruption has also been proposed to contribute to the onset and progression of certain neurodegenerative diseases. Based on multidimensional evidence from sleep parameters, clock gene expression, longitudinal follow-up data, and genetic variation, the relationships among abnormal light exposure, circadian disruption, and neurodegenerative diseases have received increasing attention. However, most current evidence is derived from observational studies and animal models, and the causal chain remains to be fully established. Further studies conducted under conditions that more closely reflect real-world clinical settings are therefore needed to validate these associations.

Future studies should further characterize the upstream and downstream molecular mechanisms and neural circuits through which BL affects central and peripheral biological clocks, and clarify the mediating role and causal contribution of circadian disruption in BL-induced mood disorders and neurodegenerative changes. In parallel, high-quality population-based cohort and interventional studies are needed to quantify the health effects of different light intensities, spectral compositions, exposure timings, and exposure durations. From a translational perspective, optimization of spectral composition and lighting strategies may help identify technical approaches to reduce the potential circadian-disrupting effects of electronic screens and artificial lighting. Furthermore, integrated strategies combining light management, sleep interventions, pharmacological approaches, and behavioral interventions may help restore circadian homeostasis, thereby providing new approaches for the prevention and management of mood disorders and neurodegenerative diseases, while also offering a scientific basis for improving environments characterized by excessive BL exposure and promoting healthier daily rhythms.

## Figures and Tables

**Table 1:** Experimental modeling of different rhythmic disruptions.

Type	Example	Specificities	Validation	References
Gene editing	*Clock*Δ19 *Bmal1-/-* *CRY1/2 Double Knockout*	Dominant negative mutation Complete rhythm deficit Extended free-run cycle (>26 h)	Fragmentation of activity rhythms Glucose metabolism disorder Destruction of sleep structure	[37,38]
Behavioral interventions	Restrictive feeding Sleep deprivation	Fixed 4-h daily feeding window (ZT6-ZT10) Multi-Platform Water Environment	Peripheral organs (liver) are uncoupled from SCN rhythms Dysrhythmia of inflammatory factors	[39]
Pharmacological Induction	Melatonin Caffeine	Inversion injection Caffeine added to drinking water	Endocrine dysfunction Decreased amplitude of body temperature rhythms	[40]
Other models	Social Jet Lag	Weekend LD 12:12 alternating with weekday LD 8:16	Weekend late-night behavior	[41]

Note: The table describes models of rhythm disruptions in different animal experiments. It describes the characteristics and application aspects of gene editing models, behavioral intervention models, drug-induced models and other modeling modalities.

**Table 2:** Role of different wavelengths of BL.

	Typical Wavelength	Biological Effect			Applications or Risks	Reference
		Target Point	Effect	Mechanisms		
Short-wave BL (400–450 nm)	420–440 nm: Peak uptake: the secondary sensitive region of the dark retinal protein OPN4	Retinal damage ^1, 2^	Induces ROS generation (significantly higher than long-wavelength light)	Mitochondrial complex III electron leakage and oxidative stress	**Risk**: Accelerated retinal photochemical damage and RPE degeneration	[121,122,123]
		Lens tissue ^1^	Promotes lens opacity and photochemical alterations	Direct photon absorption by lens structural proteins	**Risk**: Contribution to cataract development in mammalian lens models	[124]
Medium-wave BL (460–490 nm)	460–470 nm: OPN4 maximum absorption peak λmax = 480 nm 475–490 nm: Morning Natural Light Key Ingredients 475–490 nm: LED screen main emission band	Rhythm phase reset ^3^	CT14 irradiation induces robust circadian phase delay	Activation of non-visual phototransduction and ipRGC downstream pathways	**Jet lag therapy**: 460 nm light/pulsed light accelerates rhythmic phase adjustments	[125]
		Increased alertness ^3^	Improves cognitive performance and reaction speed	Enhanced central nervous system arousal and wakefulness	**Side effects**: Increased risk of circadian and metabolic disruption with nocturnal exposure	[56,126]
		Metabolic regulation ^3^	Disruption of central and peripheral clock synchrony	Alteration of metabolic rhythm and autonomic nervous axis signaling	**Metabolic risk**: Adverse metabolic and cardiovascular consequences of circadian misalignment	[126,127]
Long-wave BL (490–500 nm)	490–500 nm: Approaching the blue-green light boundary	Emotional regulation ^2, 3^	Improvement in mood and mitigation of depression-like behavior	Activation of light-sensitive neural circuits and brain reward/emotion pathways	**Brain health**: Timing of light exposure affects mood and neural circuits	[128]
		Sleep effects ^2, 3^	Wavelength-dependent alterations in sleep architecture and EEG power density	Weaker melatonin suppression compared to shorter peak wavelengths (e.g., 460 nm)	**Sleep impact**: Wavelength-dependent effects of evening light exposure on sleep structure	[97,129]
		Neural/Brain recovery ^3^	Modulates white matter water diffusion and microstructural recovery	Non-visual light therapy targeting brain microstructural recovery	**Medical application**: Blue-light therapy following mild traumatic brain injury (mTBI)	[130]

Note: Typical wavelengths, biological effects and applications, and risks of short, medium, and long wave BL. ^1^ in vitro study, ^2^ animal study, ^3^ human study.

## Data Availability

No new data were created or analyzed in this study. Data sharing is not applicable to this article.
